# Targeting the Human T-Cell Inducible COStimulator Molecule with a Monoclonal Antibody Prevents Graft-vs-Host Disease and Preserves Graft vs Leukemia in a Xenograft Murine Model

**DOI:** 10.3389/fimmu.2017.00756

**Published:** 2017-06-30

**Authors:** Aude Burlion, Simon Brunel, Nicolas Y. Petit, Daniel Olive, Gilles Marodon

**Affiliations:** ^1^Sorbonne Universités, UPMC Université Paris 06, CIMI-PARIS (Centre d’Immunologie et des Maladies Infectieuses), INSERM U 1135, CNRS ERL 8255, Paris, France; ^2^Centre de recherche en Cancérologie de Marseille, INSERM U1068, CNRS U7258, Aix Marseille Université, Institut Paoli – Calmettes, Marseille, France

**Keywords:** graft-vs-host disease, graft vs leukemia effect, antibodies, monoclonal, mice, NSG, ICOS, CD278

## Abstract

**Background:**

Graft-vs-host disease (GVHD) is a major complication of allogenic bone marrow transplantation (BMT). Targeting costimulatory molecules with antagonist antibodies could dampen the excessive immune response that occurs, while preserving the beneficial graft vs leukemia (GVL) of the allogeneic response. Previous studies using a mouse model of GVHD have shown that targeting the T-cell Inducible COStimulator (ICOS, CD278) molecule is beneficial, but it is unclear whether the same applies to human cells.

**Methods:**

Here, we assessed whether a monoclonal antibody (mAb) to human ICOS was able to antagonize the costimulatory signal delivered *in vivo* to human T cells. To test this hypothesis, we used a xenogeneic model of GVHD where human peripheral blood mononuclear cells were adoptively transferred in immunocompromised NOD.SCID.gc-null mice (NSG).

**Results:**

In this model, control mice invariably lost weight and died by day 50. In contrast, 65% of the mice receiving a single injection of the anti-hICOS mAb survived beyond 100 days. Moreover, a significant improvement in survival was obtained in a curative xeno-GVHD setting. Mechanistically, administration of the anti-hICOS mAb was associated with a strong reduction in perivascular infiltrates in liver and lungs and reduction in frequencies and numbers of human T cells in the spleen. In addition, the mAb prevented T-cell expansion in the blood during xeno-GVHD. Importantly, GVHD-protected mice retained the ability to control the P815 mastocytoma cell line, mimicking GVL in humans.

**Conclusion:**

A mAb-targeting human ICOS alleviated GVHD without impairing GVL in a xenograft murine model. Thus, ICOS represents a promising target in the management of BMT, preventing GVHD while preserving GVL.

## Introduction

Graft-vs-host disease (GVHD) is a major complication of allogeneic bone marrow transplantation (BMT), used to treat hematological malignancies. However, the allogeneic T-cell response is often necessary to eliminate residual malignant cells, an effect referred to as graft vs leukemia (GVL). Thus, the “dilemma” for efficient BMT resides on the control of a low-grade GVHD with a sufficient allogeneic response to preserve GVL (1). Targeting costimulatory molecules such as CD28 with monoclonal antibodies (mAbs) has the potential to inhibit T-cell activation and, thus, be favorable for GVHD control (2). Among other costimulatory molecules of the CD28 immunoglobulin superfamily, the Inducible T-cell COStimulator (ICOS, CD278) has been shown to be an important mediator of T-cell proliferation and survival (3–6). ICOS-ligand (ICOS-L, CD275, B7-H1, or B7RP-1) is the only known ICOS partner, constitutively expressed by B cells, and can be induced on macrophages and dendritic cells (4, 7). ICOS/ICOS-L interaction has been described to play an important role in T-cell activation and function such as cytokine production (8) and antibody class switching by B cells (9, 10). Many studies have reported a crucial role for ICOS in numerous pathologies. For instance, ICOS/ICOS-L interaction is critical for T helper cell-mediated lung mucosal inflammatory responses (5) and for collagen-induced arthritis (11, 12). In transplantation, inhibition of the ICOS/ICOS-L interaction also allows better allograft survival (13, 14). Other studies have evaluated the efficacy of ICOS blockade in mice models of GVHD. For instance, transplantation of ICOS^−/−^ T cell or anti-ICOS mAb leads to a reduced acute GVHD (15, 16). However, others have found more severe GVHD when ICOS^−/−^ CD8^+^ T cells were transferred (17, 18). Thus, the precise role of ICOS in murine GVHD is still controversial. Furthermore, the impact of ICOS blockade on GVL is currently unknown.

To address this question in a human setting, we used the transfer of human peripheral blood mononuclear cells (PBMC) in irradiated immunocompromised NOD.SCID.γc^−/−^ (NSG) mice that lacks T, B, and NK cells, as described earlier (19). Activation of human cells in this xenogeneic and lymphopenic environment then leads to GVHD symptoms, such as weight loss, infiltration of human cells in various tissues, and ultimately death. Using a model of xeno-GVHD in NSG mice that we previously established (20), we assessed here whether a murine mAb to human ICOS, previously reported as an antagonist of ICOS/ICOS-L interaction *in vitro* (21), would prevent GVHD while preserving GVL.

## Materials and Methods

### Antibodies

The 314.8 mouse anti-human ICOS mAb was produced as ascites and purified by protein A binding and elution with the Affi-gel Protein A MAPS II Kit (Bio-rad). Mouse IgG1 isotype control (MOPC-1 clone) was purchased from Bio X Cell (West Lebanon, NH, USA).

### Preparation of Human Peripheral Blood Mononuclear Cells (huPBMC)

Human peripheral blood mononuclear cells were collected by Etablissement Français du Sang from healthy adult volunteers after informed consent in accordance with the Declaration of Helsinki and isolated on a Ficoll gradient (Biocoll). Cells were washed in PBS 3% FCS and diluted at the appropriate cell concentration in 1× PBS before injection into mice.

### Mice and Xeno-GVHD Model

All animals used were NSG (NOD.SCID gamma-c^−/−^ H2-K^d^, H2-D^b^) mice (stock ≠005557) purchased from the Jackson laboratory (USA). Mice were bred in our animal facility under specific pathogen-free conditions in accordance with current European legislation. All protocols were approved by the Ethics Committee for Animal Experimentation Charles Darwin (Ce5/2012/025). For xeno-GVHD induction, 7–12-weeks-old female mice were irradiated (2 Gy) and engrafted the same day with 2.10^6^ huPBMC by retro orbital injection. Anti-hICOS antibody or control isotype was injected intraperitoneally at various doses (see figure legends). General conditions, body weight and survival of mice were monitored every 2 days to evaluate GVHD progression. Mice were euthanized when they reached 80% of their initial weight or exhibited signs of GVHD, such as hunched back, ruffled fur, and reduced mobility.

### Phenotypic Analysis by Flow Cytometry

Spleen cells were isolated by mechanical dissociation. Splenocytes were washed with 1× PBS 3% FCS and stained with human-specific antibodies used for flow cytometry: phycoerythrin (PE)-CF594-labeled CD45 (HI30 clone), allophycocyanin (APC)-H7-labeled CD8 (SK1 clone), and Alexa-fluor 700-labeled CD45RA (HI100) purchased from Becton Dickinson (San Jose, CA, USA); phycoerythrin-cyanin7-labeled CD3 (SK7 clone), peridinin chlorophyll-labeled CD4 (clone RPA-T4 clone), and APC-labeled ICOS (C398.4A clone) purchased from Biolegend (San Diego, CA, USA); and eFluor 450-labeled Ki67 (clone 20Raj1) and PE-labeled ICOS-L (clone MIH12) purchased from eBioscience (San Diego, CA, USA). Cells were stained during 20 min at room temperature and were washed with 1× PBS 3% FCS before acquisition on LSRII cytometer (Becton Dickinson, San Jose, CA, USA). Data were analyzed using the FlowJo software (TreeStar, Ashland, OR, USA).

For cytokine production analysis, freshly harvested splenocytes were stimulated 4 h with PMA (50 ng/ml) and ionomycin (500 ng/ml), both from Sigma (Saint-Louis, MO, USA) prior to extracellular staining. Cells were then fixed and permeabilized using the Cytofix/Cytoperm kit according to manufacturer’s instructions (eBioscience) and stained with CD45, CD4, CD8, and anti-human IFNγ-FITC, antihuman TNFα-PE-Cy7, and anti-human IL-2-PE (all from eBioscience). Because PMA/iono stimulation induced massive reduction of CD4 expression but not CD8 expression, we were unable to analyze cytokine production on gated CD4^+^ cells. Proportion of CD8^+^ cells producing one, two, or three cytokines was determined using boolean gating in FlowJo. For cell counting, 100 µl of blood was collected on 20 µl of heparin (Panpharma, Luitré, France). Extracellular staining for CD45, CD3, CD4, and CD8 was performed on total blood. Red blood cells’ lysis was performed using the Beckman Coulter T q-prep automaton. Then, 100 µl of fluorescent beads (Stem kit Beckman Coulter) were added and samples were run on LSRII cytometer. The absolute count of different populations was determined using the following formula [(cell count × beads concentration)/beads count].

### Histological Analysis

Liver, lungs, and rectum were embedded in OCT embedding matrix (CellPath Ltd., Mochdre, UK) and immediately frozen in liquid nitrogen. Organs were further sectioned (6 µm thick) for hematoxylin–eosin staining. Slides were coded without reference to prior treatment and examined in a blinded fashion for quantification. For liver, localized infiltrates were numbered and normalized with vessel number. For lungs, degree of infiltrate was scored according to a scale going to 0 (=no infiltrate) to 5 [=field covered by mononuclear cells (MNC)]. Histological images were acquired under an Olympus CK2 microscope (Shinjuku, Tokyo, Japan) plus Moticam 2300 camera and analyzed using the Motic Software (Motic Asia, Hong Kong). For immunofluorescence staining, slides were fixed in acetone prior to staining with DAPI and antihuman PE-labeled CD3 (UCHT1 clone; Immunotech Inc., QC, Canada). Slides were visualized with a Leica DMI 6000B fluorescence microscope, and pictures were acquired using the Leica CTR 6500 camera (Leica, Wetzlar, Germany).

### GVL Model

We chose the H2-K^d^ P815 cell line so that GVHD and GVL would target the same murine H2-K molecule. The P815 mastocytoma cell line was transduced with a lentiviral vector containing reporter genes coding for green fluorescent protein (GFP) and firefly luciferase (Luc) under the control of the phospho-glycerate kinase promoter (PGK-Luc-2A-GFP), produced in the laboratory, as described (22). After transduction, individual clones with medium GFP expression were sorted into 96-well plates using an FACS ARIA (Becton Dickinson, San Jose, CA, USA). Stable and high GFP-expressing clones were collected and frozen after 27 days of culture. Female NSG mice were irradiated (2 Gy) and injected with 2.10^6^ PBMC in 100 µl of 1× PBS in the retro orbital sinus. The next day, 2,000 P815-Luc-GFP cells were injected in the opposite retro orbital sinus and with 50 µg of the 314.8 mAb or isotype control i.p. For tumor growth monitoring, mice were injected i.p. with 3 mg of luciferin (Promega, Madison, WI, USA). Ten minutes after injection, mice were anesthetized with isofluorane and placed into the Xenogen IVIS bioluminescence imaging system (Caliper, MA, USA) in a supine position. Acquired images were analyzed using the Living Image software (Caliper Life Sciences, Inc.).

### Statistical Analysis

All statistical tests were performed using the Prism software v6.0h for Mac (Graph Pad Inc., La Jolla, CA, USA). To compare ranks between two groups, the *p* value was calculated with a non-parametric two-tailed Mann–Whitney *t*-test. Survival analyses were performed with a log-rank (Mantel–Cox) test. Tumor growth analysis was performed by non-linear regression using the exponential growth model. The *p* values of these tests are indicated on each panel. Statistical power of the analyses (alpha) was arbitrarily set at 0.05. No *a priori* test was performed to adequate the number of samples with statistical power.

## Results

### ICOS Is a Relevant Target in Xeno-GVHD

**Figure 1:**
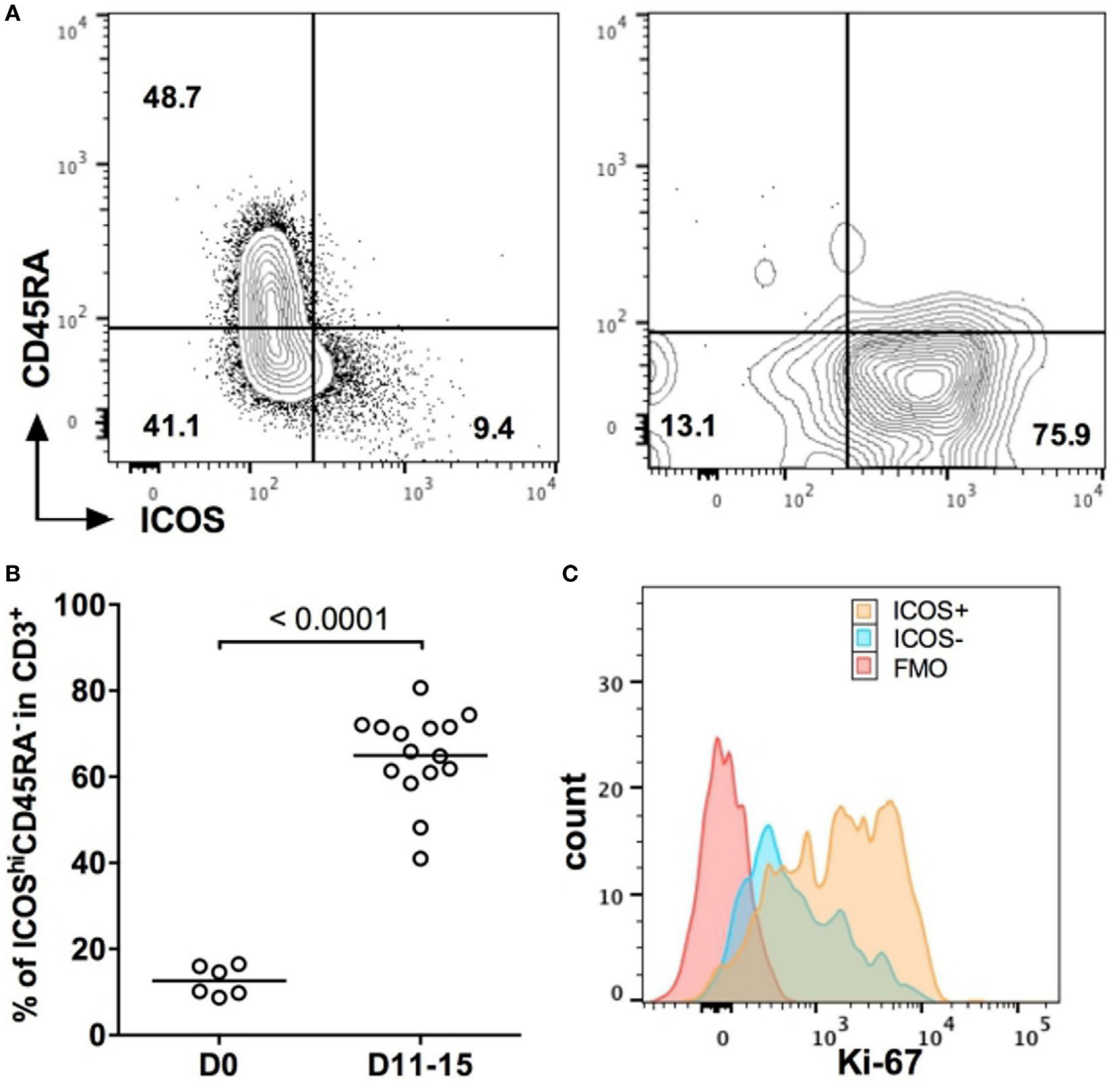
ICOS is a relevant target in xeno-graft-vs-host disease. **(A)** Representative profiles of ICOS/CD45RA expression on human CD3^+^ T cells in the blood before (D0) and 11 days (D11) after peripheral blood mononuclear cell (PBMC) injection into NSG mice. **(B)** Frequencies of ICOS^hi^ CD45RA^−^ population in CD3^+^ human T cells before injection (D0) and in the blood of NSG mice 11–15 days after injection (D11–D15). Results are compiled from two independent experiments, and each symbol represents an individual mouse or single healthy donor. The means for each group are represented by the horizontal line. **(C)** Representative profile of Ki-67 expression for ICOS^−^ and ICOS^+^ CD3^+^ T cells in the blood of NSG mice at D15 post-PBMC transfer. FMO = Fluorescence minus one control (all markers except Ki-67).

To investigate whether ICOS would represent a potential target for GVHD immunotherapy, we first monitored ICOS expression on human T cells before and after their injection in NSG mice. Before injection, CD3^+^ T cells from freshly isolated PBMC were divided into two populations according to the ICOS and CD45RA expressions: naïve T cells expressing CD45RA were mostly ICOS^lo^, whereas ICOS^hi^ cells were mostly CD45RA^−^ (Figure 1A). Eleven days after injection, CD45RA^+^ cells almost completely disappear and we observed a massive upregulation of ICOS on human T cells (Figure 1A). Overall, ICOS^hi^CD45RA^−^ cells represented 12.6 ± 3.4% of total CD3^+^ T cells before injection, whereas they represented 65 ± 10.3% of all human T cells in the blood 11–15 days after injection (Figure 1B). Moreover, expression of ICOS on CD3^+^ T cells was associated with higher levels of expression of Ki-67 (Figure 1C), a marker of cell proliferation. These results show that human T cells upregulated ICOS and proliferated during the initial stage of xeno-GVHD. This indicates that targeting ICOS with an antagonist antibody might be a relevant strategy to dampen xeno-GVHD.

### Anti-hICOS mAb Prevents and Treats Early but Not Established Xeno-GVHD

**Figure 2:**
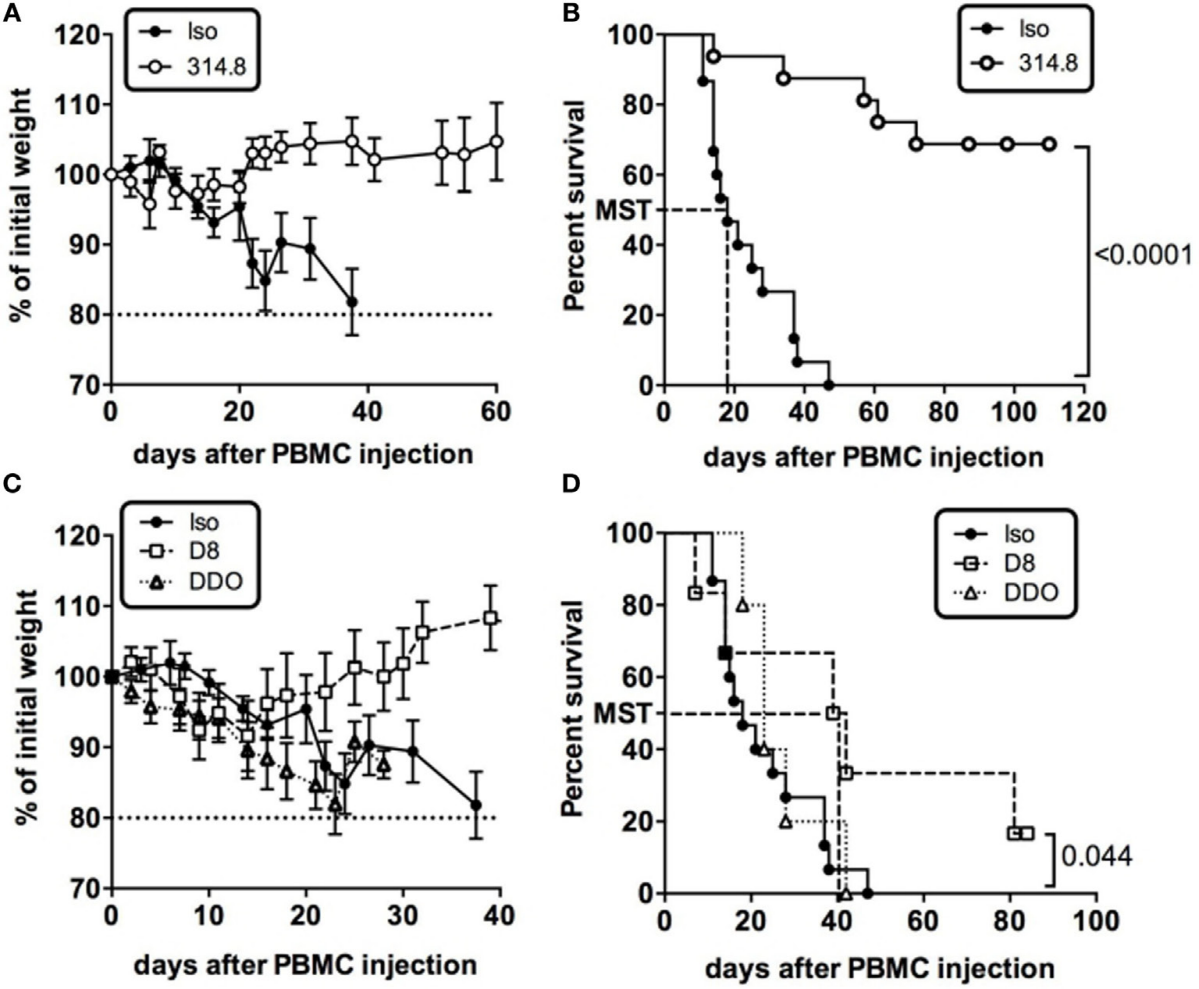
Anti-hICOS monoclonal antibody (mAb) prevents and treats early but not established xeno-graft-vs-host disease. **(A)** Percentage of initial weight is shown for NSG mice having received 500 µg of anti-ICOS antibody (314.8 clone, *n* = 23) or 500 µg of control isotype (mouse IgG1, *n* = 20) i.p. with 2.10^6^ human unfractionated peripheral blood mononuclear cells (PBMC). **(B)** Survival curves of NSG mice injected with the 314.8 (500 µg, *n* = 23) or isotype control mAbs (*n* = 20). MST = median survival time. **(C)** Percentage of initial weight is shown for NSG mice having received 500 µg of anti-ICOS antibody (*n* = 23) or isotype control mAb injected at day 8 after PBMC (D8, *n* = 6) or when mice reached 85% of their initial weight, which we referred as to the day of disease onset (DDO, *n* = 5). **(D)** Survival curves of NSG mice having received 500 µg of 314.8 at D8 and at DDO. MST, median survival time. Results are compiled from two to four independent experiments.

We thus performed experiments to determine the impact of a murine anti-hICOS mAb on xeno-GVHD. On the same day, NSG mice received fresh huPBMC and a single i.p. injection of 500 µg of the 314.8 anti-hICOS mAb or IgG1 isotype control. Ten days after, control mice exhibited symptoms of GVHD with hunched back and anemia. They also rapidly lost weight, whereas anti-hICOS-treated mice sustained their weight (Figure 2A). Sixty-five percent of the mice injected with the anti-hICOS mAb survived GVHD for more than 100 days compared to control mice, which were all dead by day 50 (Figure 2B). The median survival time of the control group was 18 days, whereas it was undefined for the anti-hICOS-treated groups since less than 50% of the mice succumbed from the disease. Additional experiments showed that this protection was also maintained at lower doses of anti-hICOS down to 10 µg per mouse (Figure S1 in Supplementary Material). Given the efficacy and apparent complete prevention of GVHD in a high proportion of mice, we questioned whether this mAb would also be effective in a curative setting. To address this, the anti-hICOS was injected on the day mice reached 85% of their initial weight, corresponding to the day of disease onset (DDO). Treated mice kept on losing weight and died at a similar rate than the controls (Figures 2C,D), showing that a single injection of a high dose of the mAb was not sufficient to impact survival in established GVHD. We thus assessed another curative approach and delayed anti-hICOS injection until D8 after PBMC injection. In this protocol, treated mice maintained their weight at initial values up to day 30 compared to the control group, which progressively lost weight (Figure 2C). This protocol was associated with the doubling in the median survival time from 18 days in the control group to 40.5 days in the D8-treated group (Figure 2D). Altogether, these results showed that a single injection of the anti-hICOS mAb could prevent and treat early but not established xeno-GVHD.

### Anti-hICOS mAb Prevents Human Mononuclear Cell Infiltration in Liver and Lungs

**Figure 3:**
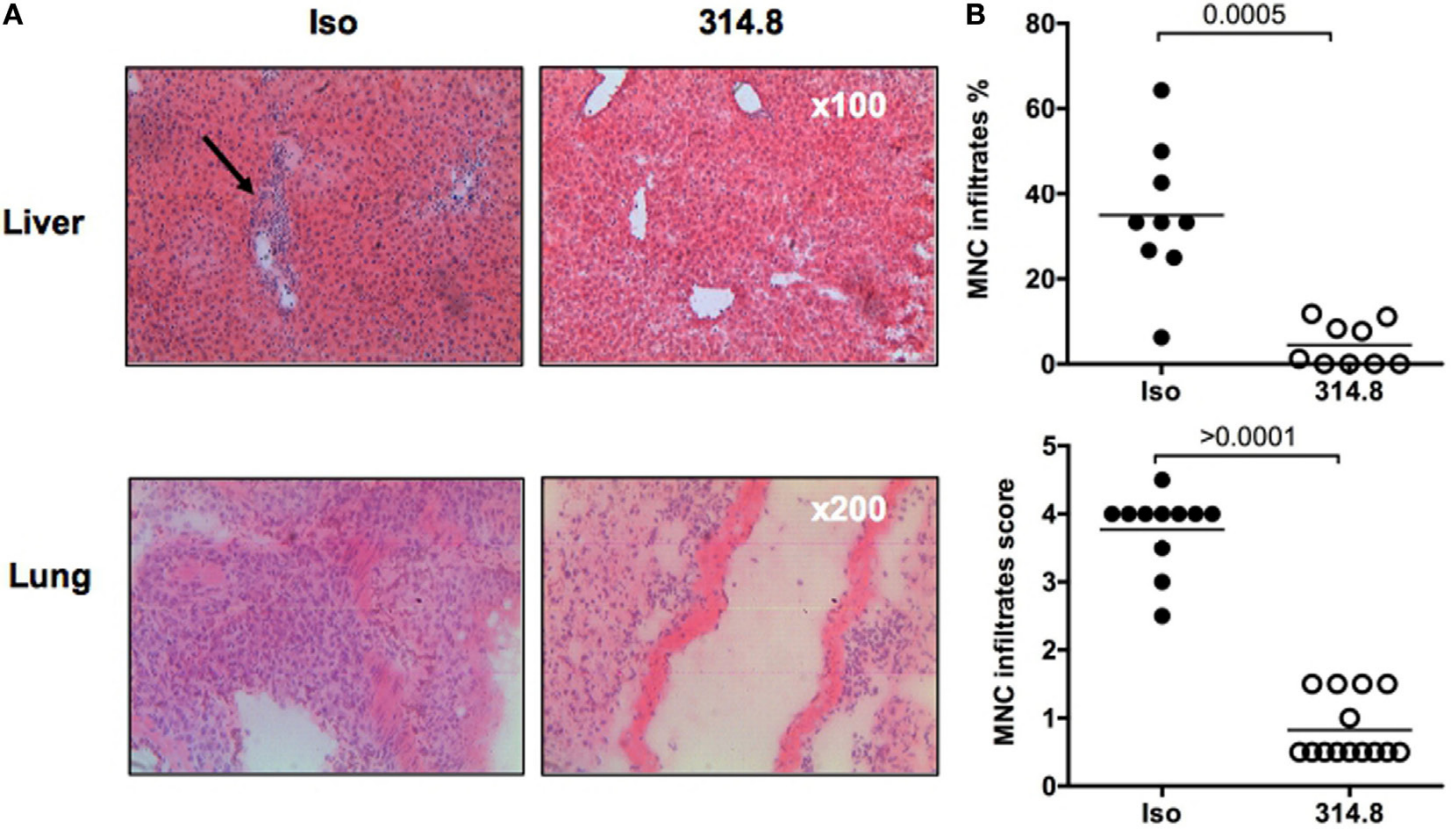
Anti-hICOS monoclonal antibody reduces human mononuclear cell (MNC) infiltration in liver and lungs. **(A)** 21 days after peripheral blood mononuclear cell injection, controls and 314.8-treated (500 µg) mice were euthanized. Livers and lungs were frozen and sectioned for hematoxylin–eosin staining. Arrow indicates infiltration surrounding the blood vessel. Magnification is indicated in the bottom right corner of each photography, representative of the entire group. **(B)** The MNC infiltrate was scored for each group and organ, according to the method described in Section “Materials and Methods.” The score means of each group are indicated by the horizontal line. Each dot is a count from one slide. Three mice per group were analyzed.

Next, we sought to understand the mechanism underlying the prevention of GVHD by the anti-hICOS mAb. We performed hematoxylin–eosin staining in the lungs, liver, and rectum of mice to analyze MNC infiltrates. In isotype control treated mice, numerous infiltrates were found and localized around blood vessels in the liver, covering most of the tissue section in the lung (Figure 3A). As expected, these infiltrates were mainly composed of human T cells (Figure S2 in Supplementary Material) and were visibly reduced in anti-hICOS-treated mice (Figure 3A). Upon blinded evaluation, we observed that the percentage of vascular structure infiltrated by MNC in the liver and the infiltration score in the lung were significantly reduced in anti-hICOS-treated mice (Figure 3B). Interestingly, neither tissue abnormalities nor signs of infiltration were observed in the rectum of NSG mice of both groups (Figure S3 in Supplementary Material), in agreement with the observation that the gamma-c molecule plays an important role in human T-cell infiltration in the intestine (23). Thus, protection conferred by anti-hICOS treatment was associated with lower human MNC infiltrates in xeno-GVHD-targeted organs, ensuring tissue integrity.

### Anti-hICOS Antibody Prevents Human T Cells’ Expansion but Not Effector Cytokine Production

**Figure 4:**
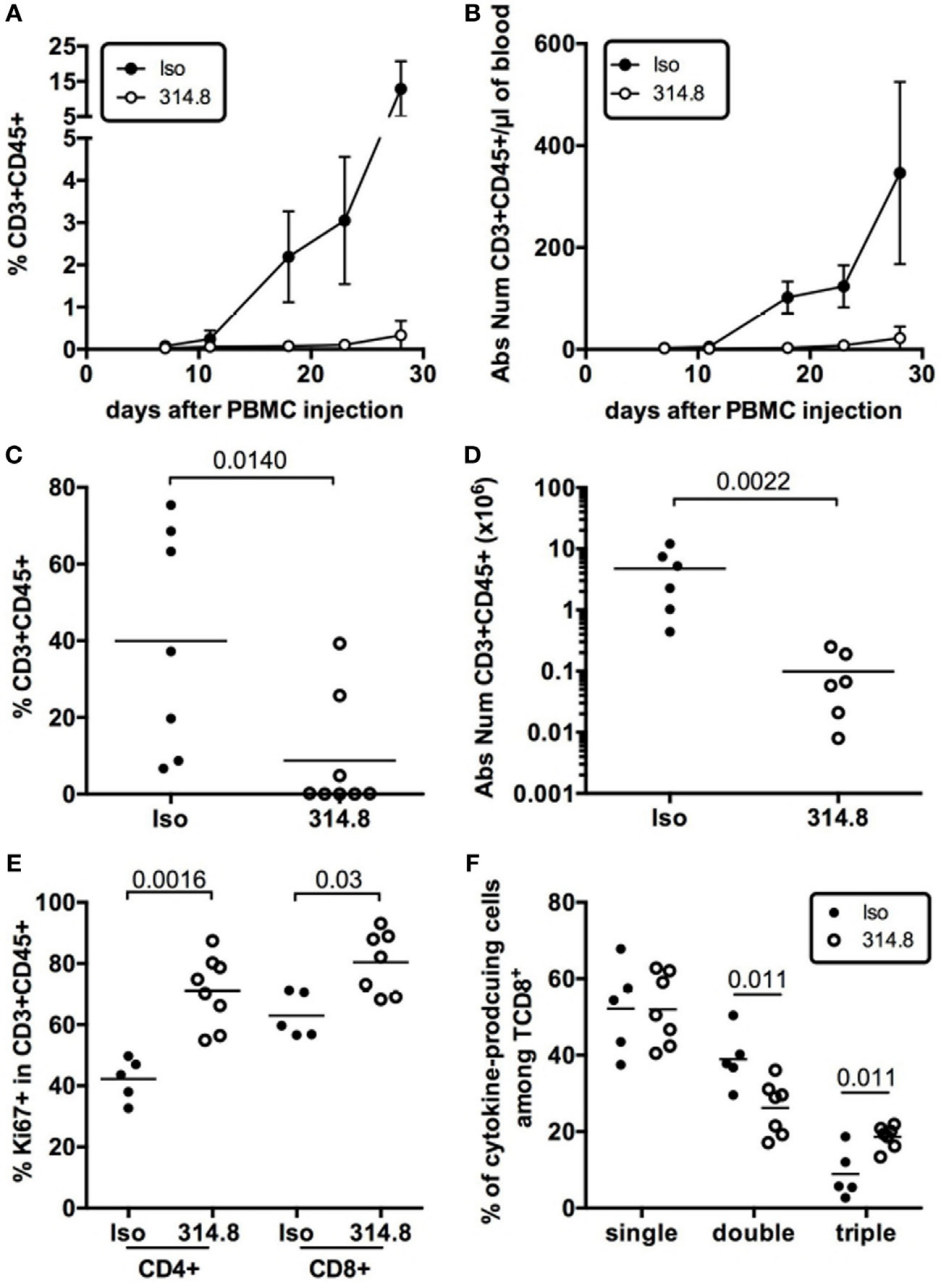
Anti-hICOS antibody prevents human T cells’ expansion but not effector cytokine production in NSG mice. Frequencies **(A)** and absolute numbers **(B)** of CD3^+^CD45^+^ cells in the blood of NSG mice at the indicated times after peripheral blood mononuclear cell (PBMC) injection in the indicated groups. Error bars are SD. Results are compiled from 2 to 16 mice in the Isotype control group (Iso) and from 4 to 9 mice in the 314.8-treated group (314.8). Error bars are SD. Frequencies **(C)** and absolute numbers **(D)** of CD3^+^ CD45^+^ T cells in the spleen 21 days after injection of PBMC in the indicated groups. Two outliers in the 314.8 group and one in the isotype control group were excluded from absolute number analysis based on the ROUT outlier detection method (*Q* = 0.5%) in Prism. Each dot is a mouse. **(E)** Frequencies of Ki-67^+^ cells in gated CD4^+^CD3^+^CD45^+^ or CD8^+^CD3^+^CD45^+^ T cells in the indicated groups. Each dot is a mouse. **(F)** Frequencies of CD8^+^ T cells producing one (single), two (double), or three (triple) cytokines of any combinations of IL-2, TNFα, or IFNγ after *ex vivo* restimulation of splenocytes with PMA/ionomycin. Mice were analyzed at D17 or D19 after injections. Each dot is a mouse. Results for this figure are compiled from two to three independent experiments with two to three unrelated healthy donors. Means for each group are represented by the horizontal line.

To determine the effect of the mAb on T-cell expansion during xeno-GVHD, we measured frequencies and numbers of human CD3^+^ T cells in the blood of isotype control vs anti-hICOS treated mice. Rapid expansion of human CD3^+^ T cells was observed for the control group from D10 onward, whereas this expansion was severely compromised in the anti-hICOS-treated group (Figures 4A,B). This diminished expansion in the blood translated into lower frequencies and numbers of human CD3^+^CD45^+^ cells in the spleen of anti-hICOS treated vs control mice (Figures 4C,D). These results show that the mAb had a systemic effect, affecting T-cell numbers in the liver, lungs, spleen, and blood, compatible with the prevention of GVHD. Paradoxically, human T cells in the spleen exhibited higher frequencies of Ki-67^+^ cells after anti-hICOS treatment (Figure 4E). Thus, the anti-hICOS mAb was able to reduce T-cell numbers, despite enhanced proliferation of residual T cells.

In addition to reduced T-cell numbers, another hypothesis to explain the protective effect of the anti-hICOS mAb would be that T cells lacked functional competency after treatment. To address this possibility, we monitored the production of IL-2, TNFα, and IFNγ by T cells isolated *ex vivo* from the control and the anti-hICOS treated groups. This analysis revealed no gross abnormalities in the ability of T cells to produce those cytokines. To the contrary, the frequencies of poly-functional CD8^+^ T cells able to produce all three cytokines were slightly increased in the anti-hICOS-treated group (Figure 4F). Thus, protection from xeno-GVHD was not associated with T-cell dysfunction, at least at the level of cytokine production.

### Preservation of GVL after Anti-hICOS mAb Treatment

**Figure 5:**
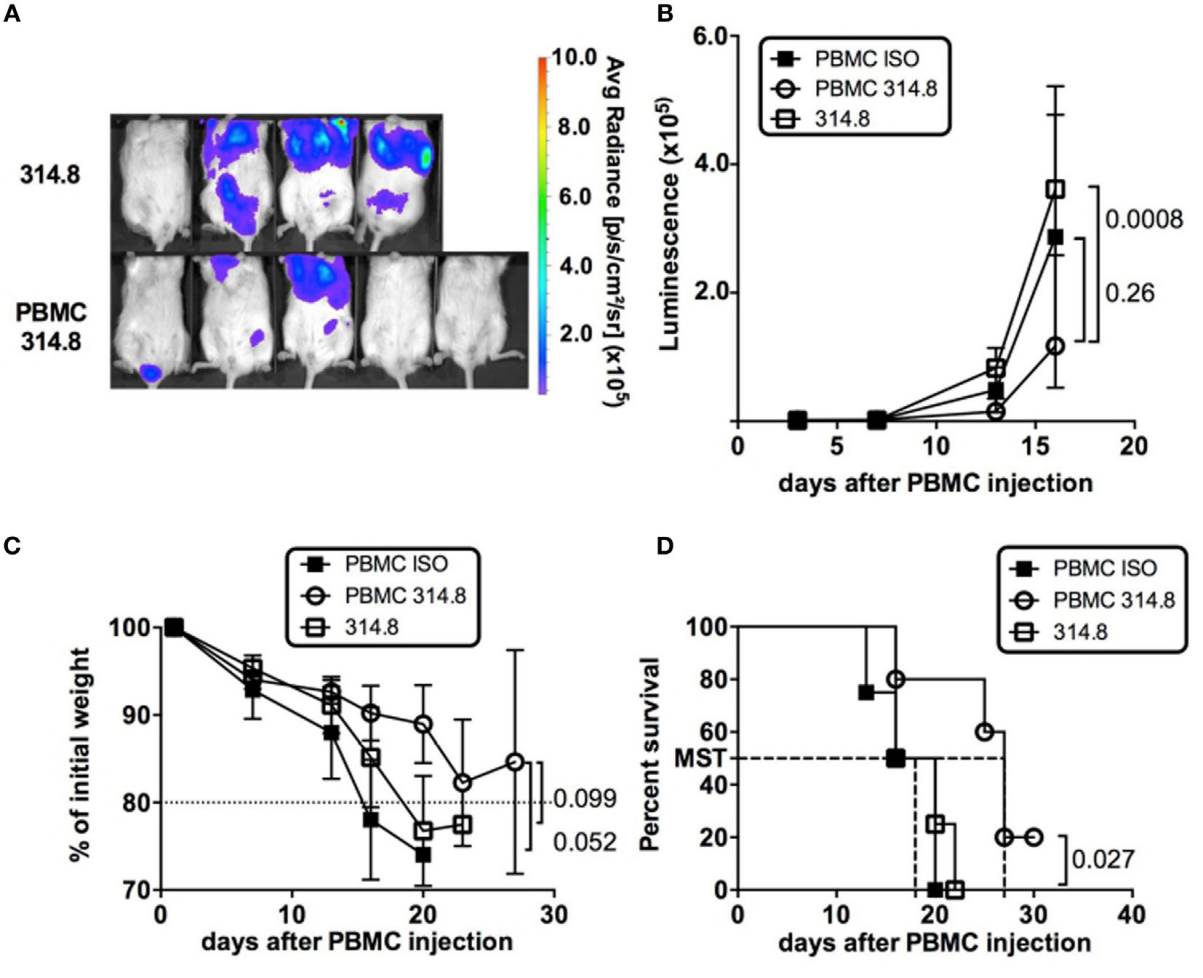
Anti-ICOS treatment preserves graft vs leukemia in graft-vs-host disease-protected mice. **(A)** P815-luciferase (Luc) bioluminescence is shown at day 16 in the indicated groups. The average radiance scale (in photons/second/cm^2^/surface) is shown. **(B)** Luminescence of P815-Luc cell line (expressed in average radiance) in NSG mice having received the treatment indicated in the legends. **(C)** Weight loss is figured as the percentage of initial weight in the indicated groups. **(D)** Survival of NSG mice in the indicated groups. Mice were entered as “dead” if they were effectively dead at the time of examination or below 80% of their initial weight. Results shown are from a total of four mice in the 314.8 only control group, five in the peripheral blood mononuclear cell (PBMC) 314.8 group, and four in the PBMC ISO group. Error bars are SEM throughout the figure.

The holy grail of BMT would be to dampen GVHD without weakening GVL responses, which are essential to prevent relapse in patients. The preserved T-cell poly-functionality and proliferative status following anti-hICOS mAb treatment prompted us to examine whether these phenotypes could be associated with a preserved GVL potential. To test the GVL capacity of GVHD-protected mice, we used the murine mastocytoma P815 cell line genetically modified to express the Luc reporter gene to follow the dissemination and growth of the tumor *in vivo* over time in live animals. To establish that PBMC in anti-hICOS-treated animals retain the ability to control P815 growth, we compared the average radiance of mice with or without PBMC. This analysis revealed that the growth of P815 with PBMC was significantly lower than in those without PBMC (Figures 5A,B), showing that the growth of P815-Luc cells was partially controlled by PBMC in anti-hICOS-treated mice. In our setting of retro orbital injections, and despite the low number of injected cells, the impact of P815 on mice survival was major since mice without PBMC lost weight and died with a median survival time of 18 days (Figures 5C,D). In contrast, animals treated with PBMC and the anti-ICOS mAb tended to lose weight at a lower rate and survived longer (median survival time 27 days) than mice without PBMC (Figures 5C,D). An additional control group (mice with PBMC and treated with an isotype control) also controlled P815 growth at similar rate than anti-ICOS-treated mice (Figure 5B). However, this group was not protected from GVHD and thus lost weight faster and died sooner than the anti-hICOS-treated group (Figures 5C,D). A similar conclusion on the efficacy of anti-hICOS-treated PBMC to delay P815 growth could be made from additional experiments in which the P815-Luc cell line was injected at later time points in GVHD-protected mice (Figure S4 in Supplementary Material). Thus, mice treated with the anti-hICOS mAb still had a GVL potential, although GVL did not fully protect mice from P815-induced death.

## Discussion

Graft-vs-host disease is a major complication of BMT, due to the allogeneic response of T cells from the donor to the recipient tissues. Although removing T cells from the graft before transplant was originally proposed (24), this is at risk of favoring relapses since donor T cells also destroy the residual disease, the GVL effect. Many groups have developed mAbs aimed at targeting costimulatory molecules involved in T-cell activation, resulting in GVHD protection in murine models (25), but some of them, such as anti-OX-40 and anti-4-1BB, have also altered GVL reaction (26, 27). Several murine studies have also shown that the ICOS pathway plays a detrimental role on GVHD, but whether GVL was maintained upon ICOS blockade was not addressed (15, 16, 28). To our knowledge, only one study reports that a humanized mAb directed against human ICOS partially protected SCID mice from a xenogeneic GVHD (29). However, the limited numbers of animals used in this study, the short duration of the follow-up, and some limitations with the CB17-SCID animal model render these data still preliminary. Most importantly, the impact of the mAb on GVL was not addressed in this study. Thus, we show here for the first time that a mAb targeting human ICOS preserved GVL in a xenogeneic graft setting, highlighting the interest of blocking this costimulatory pathway for managing BMT.

Animal models remain the only tool in which the role of ICOS on GVHD and GVL can be therapeutically addressed. Nevertheless, xeno-GVHD is a questionable model to assess human T-cell activation *in vivo* toward GVHD-targeted tissues (30). Several studies have used immunocompromised mice to address the therapeutic potential of various mAb to prevent GVHD (31–33). A xeno-GVHD model in NOG mice, a close relative of NSG mice, established that repeated injections of a humanized anti-CD26 mAb were able to diminish lethality but not weight loss associated with GVHD, in contrast to our study where both of these GHVD hallmarks were reduced (34). This study also claimed that the mAb preserved GVL using the P815 cell line implanted subcutaneously. Nevertheless, the P815 cell line might not be the optimal cell line to assess GVL potential in a xenograft setting since we show here that it is not fully rejected by huPBMC. Future studies using primary hematological malignancies might improve the relevance of xenograft studies for GVL.

The lower expansion of human T cells in the blood of anti-hICOS-treated mice suggests that ICOS/ICOS-L interaction was needed for proper expansion of human T cells, associated with GVHD severity. This would be coherent with the proposed role for ICOS for proper T-cell activation. Another hypothesis to explain reduction in T-cell numbers following mAb exposure *in vivo* would imply antibody-dependent cell-mediated cytotoxicity (ADCC). Thus, ADCC may have occurred as the results of the binding of the 314.8 murine IgG1 Fc fragment on FcRγ and the subsequent activation of phagocytosis by myeloid cells present in NSG mice. Indeed, peritoneal macrophages of NSG mice have been shown to deplete human T cells in the presence of the OKT3 mAb, showing that opsonization is active in those mice (35). Thus, the mechanism(s) by which the 314.8 antibody might have reduced human T cells in NSG mice is still undefined, probably a mix between ADCC and the physiological requirement for ICOS/ICOS-L on T-cell expansion. An alternative explanation for a reduced T-cell infiltration in the tissue would imply that the mAb has modified homing receptors’ expression on transplanted T cells. This is an attractive hypothesis since ICOS signaling in mice has been linked to the regulation of the *klf2* transcription factor, a master switch controlling proper homing of Tfh in the germinal center (36). This hypothesis remains to be formally addressed in the xeno-GVHD setting.

Despite reduced T-cell numbers, anti-hICOS-treated mice were still able to interfere with growth of the P815 mastocytoma cell line at similar rates than PBMC treated with an isotype control. The preservation of the GVL potential of anti-hICOS-treated T cells might be due to their higher proliferative status and/or their preserved capacity to produce effector cytokines. Indeed, we observed a paradoxical increase in poly-functional cells able to express IL-2, TNF, and IFNγ cytokines. High levels of IFNγ in xeno-GVHD settings are consistent with other studies (31, 32) and suggest that IFNγ might be an important effector cytokine secreted during xeno-GHVD that could have participated in the GVL effect.

As for human translation of preclinical data obtained in mice or humanized mice, it might be urgent to wait. To date, there is no clinical evidence that ICOS polymorphisms are associated with acute or chronic GVHD (37, 38), but larger studies covering the entire ICOS and ICOS-L locus in non-ethnical biased studies are warranted before any conclusions can be drawn. To our knowledge, only one study based on bioinformatics analysis found that low levels of ICOS mRNA were associated with acute GVHD (39), a result contrasting with our findings in the xeno-GVHD settings, showing massive upregulation of ICOS on transferred T cells. Thus, more robust clinical data showing that ICOS represents a biomarker of human GVHD severity are still needed before any clinical translation of our results might be foreseen.

## Ethics Statement

Blood samples were obtained from healthy volunteers from the Etablissement Francais du Sang. All human subjects gave written informed consent in accordance with the Declaration of Helsinki. This study was carried out in accordance with the recommendations of the Ethics Committee on Animal Experimentation Charles Darwin. The protocol was approved by the Ethics Committee on Animal Experimentation Charles Darwin (Ce5/2012/025).

## Author Contributions

SB and NP performed the experiments, analyzed the data, and revised the manuscript, DO contributed to essential reagents and revised the manuscript, and AB and GM designed the research, performed the experiments, analyzed the data, and wrote the manuscript.

## Conflict of Interest Statement

DO and GM held a patent on the use of anti-ICOS to treat GVHD that was licensed to GSK during the course of this study. GSK had no role in the design, analysis, or reporting of the results. All other authors declare no conflict of interest.

## Abbreviations

GVHD, graft-vs-host disease; GVL, graft vs leukemia; ICOS, inducible COStimulatory molecule; BMT, bone marrow transplantation; NSG, NOD.SCID.gc^−/−^.
