# Supplementary material for: Targeting the Human T-Cell Inducible COStimulator Molecule with a Monoclonal Antibody Prevents Graft-vs-Host Disease and Preserves Graft vs Leukemia in a Xenograft Murine Model
Source: Front Immunol. 2017 Jun 30;8:756. doi: 10.3389/fimmu.2017.00756 (PMC5491549; doi:10.3389/fimmu.2017.00756)
Supplement: Supplementary file 1 [file Presentation_1.PDF]

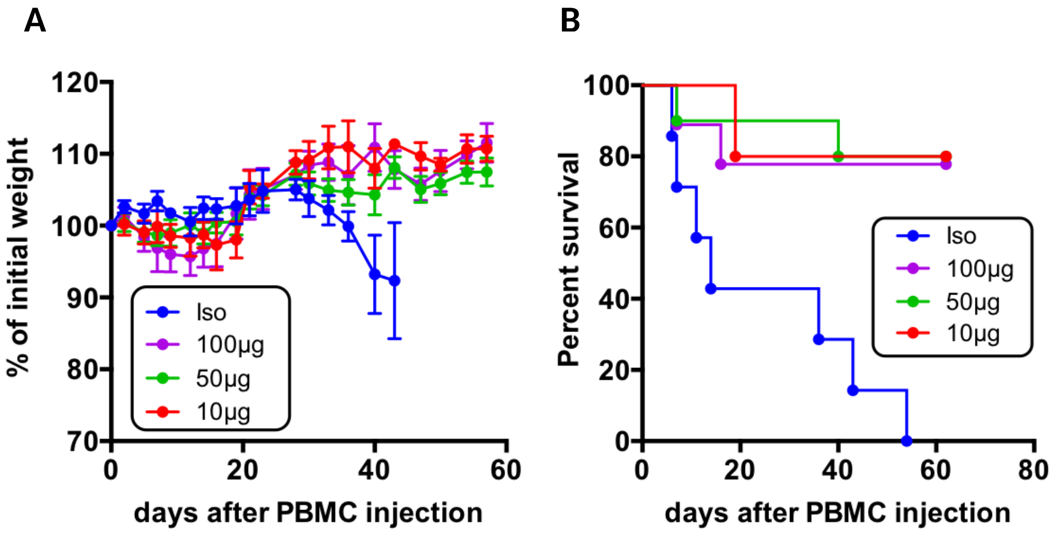

Figure S1 : anti-ICOS treatment protects from GVHD at various doses. A. Percentage of initial weight is shown for NSG mice having received indicated doses of anti-ICOS antibody (314.8 clone) i.p with 2.10^6 human unfractionated PBMCs. **B.** Survival curves of NSG mice injected with the 314.8 (10µg: n=5; 50µg: n=10; 100µg: n=9)) or isotype control antibody (n=7). Results are compiled from 2 to 3 independent experiments.

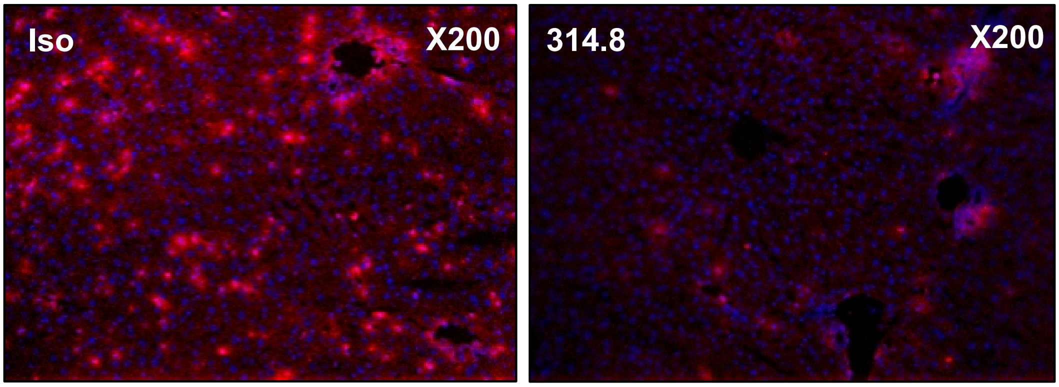

Figure S2 : **Human T cell infiltrate is reduced after 314.8 treatment in the liver.** 21 days after PBMC injection, isotype control (Iso) and 314.8 treated mice were sacrificed. Liver slides were stained with anti-CD3 PE (red) and DAPI (blue). Magnification is indicated in the top right corner of each photography, representative of the entire group.

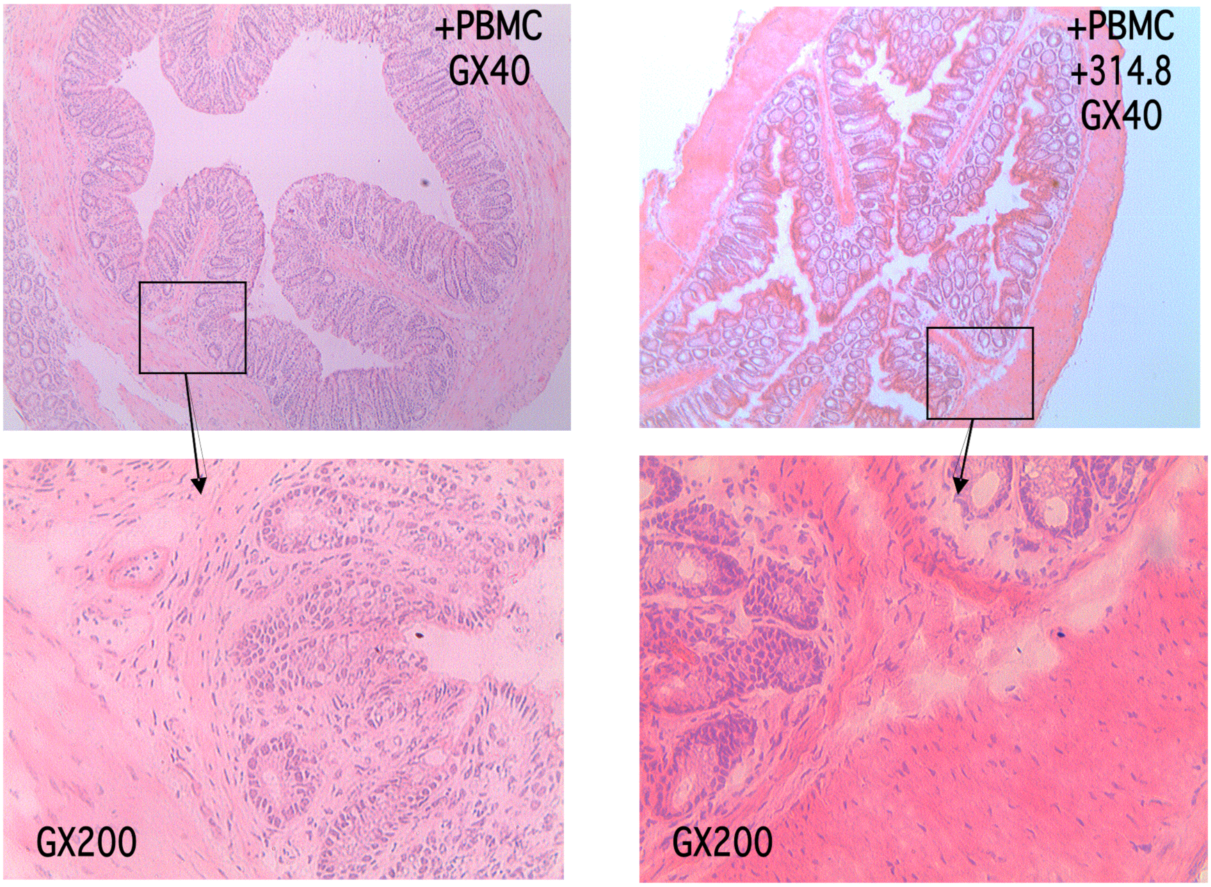

Figure S3 : No gross abnormalities of the intestinal epithelium in xeno-GVHD. Hematoxylin/Eosin staining of the rectum of NSG mice 21 days after injection with PBMC with or without anti-ICOS mAb (314.8). Magnification (GX) is indicated in each photography.

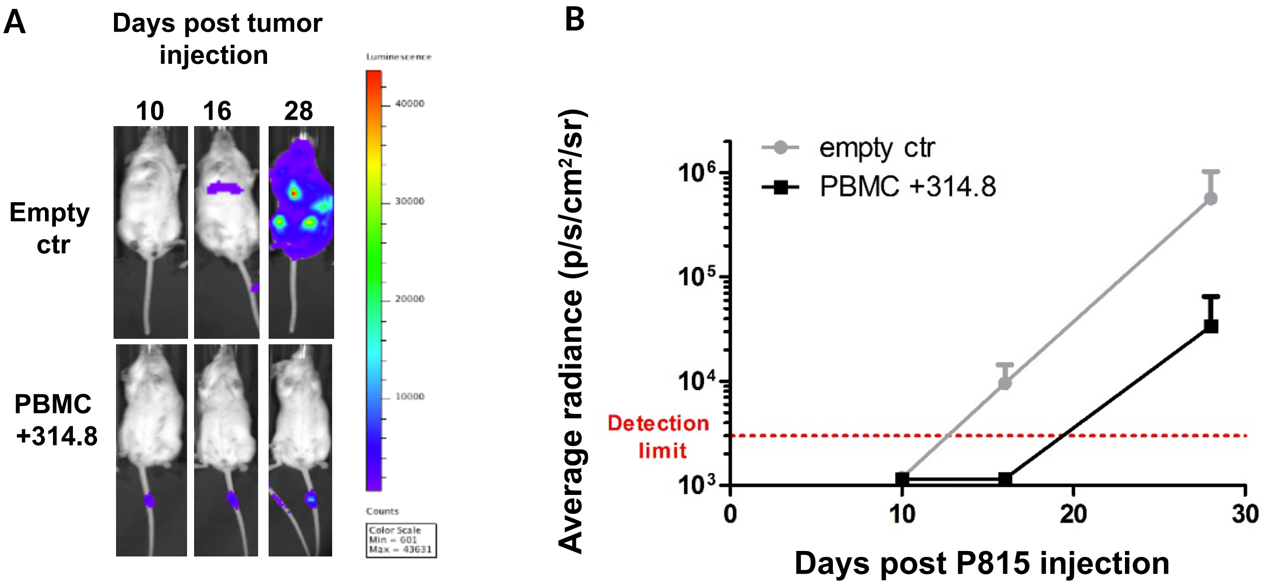

Figure S4 : GVHD-protected NSG mice are still able to elicit GVL. NSG mice were injected with PBMC and treated with the 314.8 mAb at D0. At D31 in one experiment or at D54 in another, GVHD-protected mice were injected in the tail vein with 5 000 P815-Luc. A control group of empty NSG mice (aged and sex matched) (empty ctr) were injected with the same number of PBMC from the same donor at the same time. A. Imaging of luciferase activity at the indicated days after P815 injection. B. Bioluminescence signals expressed as the average radiance in the control group without PBMC (empty ctr) or in GVHD-protected mice previously treated with PBMC and 314.8 mAb. Results are cumulative of 2 independent experiments totalizing 15 NSG mice in the treated group and 13 in the control group.
